# Factors related with public open space use among adolescents: a study using GPS and accelerometers

**DOI:** 10.1186/s12942-018-0123-2

**Published:** 2018-01-22

**Authors:** Linde Van Hecke, Hannah Verhoeven, Peter Clarys, Delfien Van Dyck, Nico Van de Weghe, Tim Baert, Benedicte Deforche, Jelle Van Cauwenberg

**Affiliations:** 1 Department of Public Health, Faculty of Medicine and Health Sciences, Ghent University, Ghent, Belgium; 2 Physical Activity, Nutrition and Health Research Unit, Department of Movement and Sport Sciences, Faculty of Physical Education and Physical Therapy, Vrije Universiteit Brussel, Brussels, Belgium; 3 Fund for Scientific Research Flanders (FWO), Brussels, Belgium; 4 Department of Movement and Sport Sciences, Faculty of Medicine and Health Sciences, Ghent University, Ghent, Belgium; 5 Department of Geography – CartoGIS, Faculty of Sciences, Ghent University, Ghent, Belgium

**Keywords:** Global positioning device, Physical activity, Sedentary time, Youth, Leisure time, Public spaces

## Abstract

**Background:**

Low physical activity levels and high levels of sedentary time among adolescents call for population wide interventions. Public open spaces can be important locations for adolescents’ physical activity. This study aimed to describe the prevalence, frequency and context of public open space visitation and to gain insight into the individual, social and physical environmental factors associated with public open space use among 12- to 16-year-old Flemish (Belgian) adolescents.

**Methods:**

Global positioning system devices, accelerometers and one-on-one interviews were used to measure location-specific activity levels, time spent at, reasons for using and accompaniment at public open spaces among 173 adolescents. Multilevel hurdle and gamma models were used to estimate the associations between the independent variables (age, gender, ethnicity, education, sport club membership and accompaniment) and the amount of time, sedentary time, light-, moderate- to vigorous- and vigorous-intensity physical activity at public open spaces.

**Results:**

Three out of four participants had visited a public open space (for recreational purposes) and participants were most often accompanied by friends/classmates. Mainly public transportation stops/stations were used, and subsequently the most reported reason for public open space use was “to wait for something or someone”. Furthermore, boys, younger adolescents, non-western-European adolescents and lower educated adolescents were more likely to use public open spaces. Additionally, boys and younger adolescents were more likely to accumulate physical activity at public open spaces. The only social environmental variable associated with time spent at public open spaces was accompaniment by siblings: adolescents spent more time at public open spaces when accompanied by their siblings.

**Conclusions:**

Public open spaces may be effective areas to promote physical activity among groups at risk for physical inactivity (i.e. low educated and non-western-European adolescents). Additionally, girls and older adolescents were less likely to visit and be physically active at public open spaces. Therefore, urban planners should consider adding attractive features, in order to encourage physical activity among girls and older adolescents at public open spaces. Furthermore, creating public open spaces that are attractive for youth of all ages could contribute to adolescents visiting public open spaces accompanied by siblings.

**Electronic supplementary material:**

The online version of this article (10.1186/s12942-018-0123-2) contains supplementary material, which is available to authorized users.

## Background

The World Health Organisation (WHO) recommends adolescents to engage in 60 min of moderate- to vigorous-intensity physical activity (MVPA) daily [1] in order to obtain health benefits such as lower risk for overweight and obesity, diabetes type 2, high blood pressure and depressive symptoms [2–5]. In addition, adolescents engaging in extended periods of sedentary time (i.e. time spent sitting or lying down at low energy expenditure [6]) are at higher risk for higher Body Mass Index (BMI), decreased fitness and lower psychosocial health [7, 8]. However, during the transition from childhood to adolescence a steep decline in physical activity (physical activity) levels [9–11] and an increase in sedentary time occurs [10, 11]. Subsequently, more than half of the adolescent population worldwide does not meet the physical activity recommendations [12–14] whilst European adolescents’ sedentary time rises to 4–8 h per day on average [15]. Furthermore, healthy behaviours concerning physical activity and sedentary time developed in adolescence are known to track into adulthood, so being sufficiently active and having low levels of sedentary time during adolescence are of high importance [16–18].

Consequently, there is a need for population wide interventions to increase adolescent physical activity levels and decrease sedentary time. In the past, mostly individually-oriented models were used for intervention development [19]. During the last decade however, a shift has been made to socio-ecological models, which emphasize the interactions between individuals and their physical and socio-cultural environment [19, 20]. The different layers of the socio-ecological model are build up around four active living domains where adolescents can be active: at home, at school, during active transportation, and during leisure time [20]. Leisure time, physical activity can occur in an organized setting such as sport clubs or in non-organized settings such as at home, in streets, parks and playgrounds. Little is known about the locations where adolescents’ non-organized leisure time physical activity (away from home) takes place and the need for more information on location-specific physical activity levels has been emphasized previously [21].

Studies in the US have shown that public open spaces (POS) are used for physical activity and recreational activities among children, adolescents and adults [22–24]. They are suitable for non-organized physical activity as they are public spaces that are freely accessible to all people, without entrance fee and present in most communities [24–26]. POS can have different appearances such as parks, playgrounds and squares, but also streets, vacant lots and parking lots. POS may be especially important for adolescents under the age of sixteen because they do not have the possibility to drive a car or moped and are, therefore, still limited in their ability to visit places located at greater distance from their residence and have to rely more on public transportation. Moreover, qualitative research has indicated that adolescents attach great importance to POS as a place where they can spend time without parental supervision or to be away from the bustle at home or school [27, 28].

On the one hand, a POS can be a suitable location for physical activity (and thereby directly increase overall physical activity levels), but on the other hand, a POS can also be a destination that adolescents can visit using active transportation (and thereby increase overall physical activity levels through active transportation) [7, 8]. This implicates that when only physical activity in POS would be considered in research (and thus not including physical activity during trips to and from POS), an underestimation of the physical activity related to POS visitation would be made. Therefore, physical activity accumulated during trips to and from POS should be included in research concerning POS use among adolescents, as these can contribute to overall activity levels even if adolescents do not accumulate physical activity in POS.

Research on POS use among adolescents is limited, but some studies have emerged recently. An Australian survey study reported almost 40% of 13-year old adolescents to have used a park at least once a week during the past 3 months. Additionally, only 12% of the adolescents reported not to have visited a park in the past 3 months [29]. Furthermore, a US study among 11- to 14-year-old adolescents using accelerometers and global positioning system (GPS) devices reported that an average of 45 min was spent daily on streets and sidewalks, 25 min at playgrounds and 17 min in parks [30]. A Danish study among 11–16-year olds with similar methodology reported lower levels of POS use, with a median of only 11.7 min/day spent at school grounds (during leisure time), 5.2 min/day in urban green space, 0.0 min/day at playgrounds and 0.0 min/day at sport facilities [31]. However, the differences in POS use between the two studies can be attributed to the fact that active transport to POS and in POS was included to calculate time spent in POS in the US study, while this was not the case in the Danish study.

However, research on the prevalence and frequency of POS visitation, the activity levels in POS, types of POS used and reasons for POS visitation among adolescents remains scarce, especially in Europe. Therefore, additional research is needed to gain insight into the prevalence and frequency of POS visitation and the activity levels in POS. Furthermore, the types of POS that are used and reasons for POS visitation should be explored in order to better understand the different aspects of (active) POS use.

As mentioned above, socio-ecological models emphasize the importance of individual-, physical- and social environmental factors to explain physical activity behaviours and sedentary time. Currently, information is lacking about factors associated with time and physical activity in POS whilst (to our knowledge) no studies have investigated the factors associated with sedentary time in POS. Because sedentary time is independently related to health problems [12, 13], identifying factors associated with sedentary time in POS is especially important. Identifying the physical and social environmental factors that could induce sedentary behaviour in POS enables to define the necessary strategies to reduce sedentary time at public open spaces. Additionally, this allows to target specific population groups at risk for sedentary time in POS.

Two Danish studies using GPS and accelerometers showed that older adolescents (mean age 14.2) spent less time [31] and less MVPA [32] at school grounds during leisure-time and more time and MVPA at sport facilities and shopping centres compared to younger adolescents (mean age 12.4) [31]. Furthermore, boys aged 11- to 16-year-old spent more time at sport facilities, accumulated more MVPA at school grounds during leisure time [31, 32] and less MVPA at playgrounds and urban green space compared to girls [32]. Furthermore, a Canadian study using GPS and accelerometers indicated that adolescents living in suburban areas performed more MVPA in POS locations such as green spaces or shopping malls compared to adolescents living in urban and rural areas, whilst no differences were found in MVPA at different POS locations according to adolescents’ Socio-Economic Status (SES) [33]. These studies indicate that individual factors such as gender and age could possibly be associated with time spent and physical activity in POS whereas, no previous research has looked into the individual factors associated with sedentary time in POS. Furthermore, it is possible that the social environment (e.g., accompaniment in POS) is associated with adolescents’ time, sedentary time and physical activity in POS, however, no studies have investigated this matter. Additionally, some physical environmental factors associated with physical activity in POS, have been identified, whereas no research has studied the associations for environmental factors with sedentary time in POS. Recent observational research has indicated that different park areas such as playgrounds, open fields or sport fields were associated with different activity levels across all age groups [34–37]. This evidence suggests associations of individual, social- and physical environmental factors with time and physical activity in POS among adolescents. However, European research is limited and additional insight is needed into the factors associated with sedentary time in POS among adolescents.

Many of the studies investigating the association between POS availability, POS use and physical activity levels have used questionnaires, geographical information systems (GIS) or audits of POS in the participants’ neighbourhood [23, 38–41], assuming that these are the locations that are most frequently used. However, adolescents may use other POS than those closest to home and, therefore, it is important to use methods such as diaries or GPS-measures that allow to investigate the locations that are actually used by the adolescents. GPS devices have been identified as more accurate compared to activity diaries [42–44]. Furthermore, when GPS devices are combined with accelerometers, it is possible to objectively measure location-specific physical activity [45].

Summarized, evidence on adolescents’ POS use and its associated individual, physical and social environmental factors is limited, with most studies originating from North-America and Australia. Only two studies originate from Europe. Most of the existing studies included measures of POS use, some included measures of physical activity in POS, whilst none included measures of sedentary time in POS. Furthermore, many studies have used methods that cannot capture the specific POS that is used. POS can be suitable locations for physical activity among adolescents. However, in order to develop interventions to promote physical activity and reduce sedentary time in POS, insight is needed into the use of POS, physical activity and sedentary time in POS and into the factors associated with POS use, physical activity and sedentary time in POS. Therefore, this study used GPS devices and accelerometers in order to (1) describe the prevalence, frequency and context (i.e. company, locations and reason) of POS visitation and (2) gain insight into the individual, social and physical environmental factors associated with time, sedentary time and physical activity in POS among 12- to 16-year-old Flemish (Belgian) adolescents.

## Methods

### Study area

The study took place in Ghent, the capital city of the province of East Flanders (Belgium). Belgium is ranked 22th in the Human Development index developed by the United Nations, with a value of 0.90 (maximum score = 1) [46]. Ghent comprises an area of 156.18 km^2^ and has 253,266 inhabitants (population density: 1622 inh/km^2^) [47, 48]. Ghent is a modern city that was founded in the eighth century at the confluence of two rivers and has a densely built historical inner city surrounded with nineteenth and twentieth century workers districts. The north of the city comprises an international harbour, whilst the south is characterised by the new train station [49].

In Ghent, the unemployment rate is 12.5, 2.0% of the population is entitled to a living wage and 18.8% is part of an ethnic-cultural minority whilst the remaining 81.2% is predominantly white [50–52]. In total, 37.0% of the inhabitants of Ghent have access to public green space (< 1 ha) within 150 m of their home and 41.9% has access to public green space (> 1 ha) within 400 m from their home [50]. Additionally, 1.8 km^2^ of the city is designated to playgrounds, woods or parks where people are allowed to play [50].

**Fig. 1:**
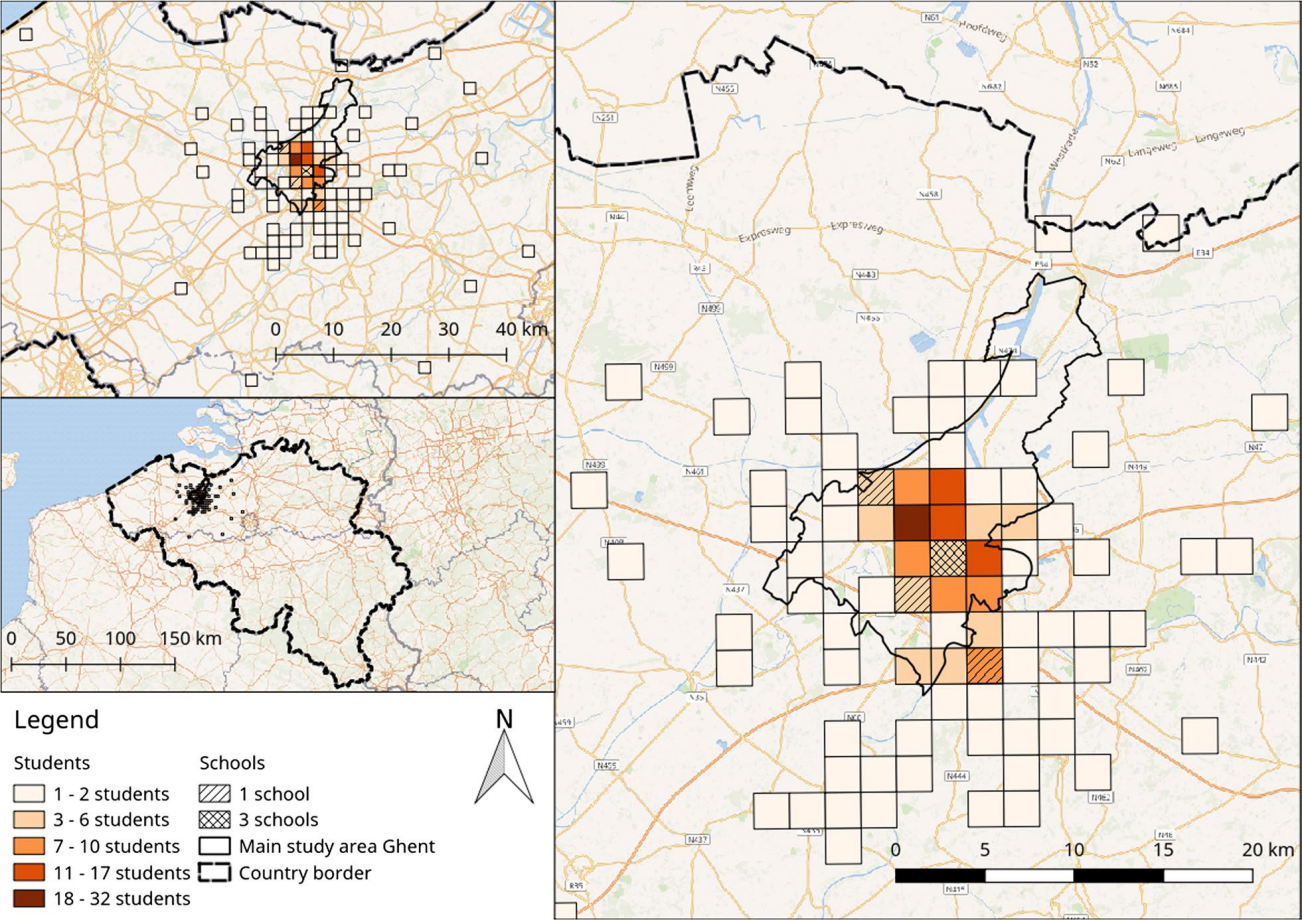
City of Ghent with location of the schools and home addresses of the participants

Four of the participating schools were located in the city centre whilst two were located in the outskirts of the city (Fig. 1).

### Participant and school recruitment

Participants (12- to 16-year-old) were recruited through schools. Before recruitment, the study design and purpose were presented in a meeting with all principals of the governmental schools located in Ghent (Flanders, Belgium). Six out of twelve schools were willing to participate. In each school at least two classes in the first to fourth grade (12- to 16-year-old) were selected by the principal or a staff member and all students from these classes were invited to participate (total of 18 classes: Additional file 1: Table S1). Participation in the study was voluntary and participants received a movie ticket as an incentive after measurements were finished.

### Study protocol

Data were collected from September to December 2015 (mean daily rainfall = 0.4 mm/day, mean daily hours of sunshine: 4.1 h/day, mean maximum temperature: 15.1 °C/day). Participating schools were visited three times by the research team. Before school visits took place, all schools were asked to distribute a parental information and consent form to all parents of students in participating classes. Parents who did not give permission for their children to participate, had to sign the parental consent form and their children could hand in these parental consent forms to the researchers at the first school visit. During the first school visit, participants were asked to read and sign a participant consent form. This approach was used because adolescents had to fill in a questionnaire on a non-sensitive topic [53, 54]. This consent procedure and the research protocol for minors were approved by the medical ethics committee of the University Hospital of Ghent University (2015/0317) referring to the privacy act of December 8th, 2012 on the protection of privacy in relation to the processing of personal data [55]. Participants received a personal ID number they could use to anonymously complete a questionnaire concerning demographics. Every participant received an accelerometer, GPS device and charger for the GPS device. The participants were given verbal and written instructions on how and when to wear the devices and how to charge the GPS overnight. All participants were asked for their phone number and those willing to give their number (n = 140; 49.5%), received two text messages daily: every morning to remind them to wear the devices and every evening to remind them to charge the GPS device.

After 4–5 days the devices were collected during the second school visit and the GPS and accelerometer data were downloaded from the devices. A web application was created to visualize the data from each participant on a map for each day the devices were worn.

**Fig. 2:**
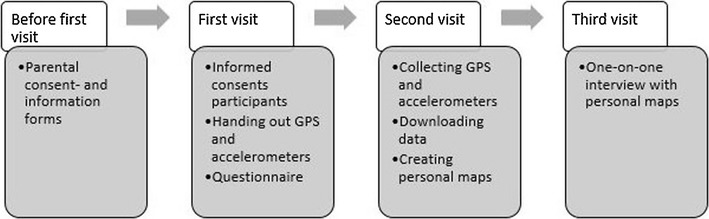
Data collection process

The third school visit comprised a one-on-one interview of 10–20 min during which the personal maps were used. During this interview, participants were asked about the reasons, activities and company in POS locations that were used. An overview of the data collection process is presented in Fig. 2.

### Measurements

#### Questionnaire

All participants were asked to complete a questionnaire that included the following questions on demographics: date of birth, place of birth, sex, address (address was used to define area of residence: rural < 300 inh/km^2^, suburban: 300–600 inh/km^2^, urban > 600 inh/km^2^ [56]), education (general, technical, vocational or arts), school grade (first to fourth year), nationality of parents, highest education of the parents (primary education, secondary education, higher education-non university, higher education-university, I don’t know [57]) and sport club membership (yes/no). Based on parental educational level, low SES was defined as: none of the parents possessed a higher education diploma whereas high SES was defined as: at least one parent possessed a higher education diploma. Based on the place of birth of the participant and the parents, a non-western-European ethnicity was defined as having at least one parent born outside of the EU15 as defined by the Flemish government [58].

#### Physical activity measurement

Physical activity was measured with ActiGraph GTX-3 devices which were worn during waking hours for 4–5 consecutive days on a belt on the right hip. The Actigraph GTX-3 is a reliable and valid instrument to measure physical activity in youth and adults [59–61]. The Actigraph accelerometer uses a piezoelectric acceleration sensor, that, when it undergoes an acceleration, produces a voltage signal that is expressed as ‘counts’ [62]. These counts were averaged in periods (called epochs) of 15 s, as recommended [63]. The counts were stored onto the accelerometer device and later on downloaded using Actilife software version 6. For each 15 s epoch, the activity level [sedentary time (e.g., watching TV while sitting down), light-intensity physical activity (LPA) (e.g., walking slowly), moderate-intensity physical activity (MPA) (e.g., walking at 7.2 km/h) and vigorous-intensity physical activity (VPA) (e.g., running) [64–66]) was determined using Evenson cutpoints (sedentary time ≤ 100; LPA > 100, < 2296; 2296 ≥ MPA < 4012, VPA ≥ 4012) [67]. Continuous periods of 60 min of zero values were classified as non-wear time and removed from the data. Only participants with at least 1 day with at least 9 h of valid data were included in the analysis [32, 68]. Thus, when GPS devices were turned off for a substantial amount of time, this could have led to that day being excluded from analysis.

#### Spatial measurements: locations

A GPS device (Qstarz BT-Q1000XT) was worn on a belt on the left hip to track the locations of the participants. The devices were configured and data downloaded using the program Q-travel. Data were logged every 30 s. Epochs of 30 s have been used successfully for GPS data processing in previous studies with adolescents [69, 70]. Additionally, Schipperijn et al. [71] showed that limited differences exist between GPS data stored at epochs of 5, 15 and 30 s and that the three data collection epochs had the same median error.

#### One-on-one interview with personal maps

**Fig. 3:**
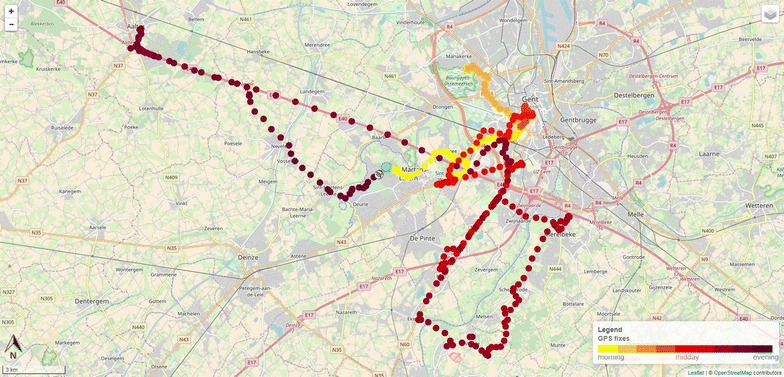
Example of a personal map

The data from the GPS devices were stored in a PostgreSQL database with PostGIS in order to visualize the visited locations of each participant in the self-made web application. The personal ID was used to log each participant in a self-made web application, where an individual map was available for each day the participant wore the devices (Fig. 3). On this individual web based map, the trip of the participant was visualized by placing a dot on the map every 30 s. Additionally, a light to dark colour scheme was used, to give an indication of the time during the day. The exact time of a point could be seen by clicking on a point. It was possible to zoom in on the map, which gave a clear overview of the locations that were visited. By using OpenStreetMap as a background layer, contextual information on the visited places of the participant could be gathered. The first week- and weekend day with complete data were selected (excluding the day the devices were handed out) and discussed with the participants. When no weekdays with complete data were available, two weekend days were selected and vice versa. For participants with only 1 day with complete data, this day was selected. For these selected days, the participants had to indicate the type of each location (e.g., school, home, a park, train station) they visited. For the locations that were classified as outdoor POS (street, shopping street/mall, square, park, outdoor sports ground/playground, parking lot, vacant lot and public transportation stop/station) three additional questions were asked: “who accompanied you here?”; “which activities did you engage in?”; “why did you choose this place?”.

The colours of the dots represent the time course of the day: every 30 s a dot was placed on the map (Temporal resolution: 30 s). Lighter colours represent the start of the day, darker colours represent the end of the day. The green arrow represents the first data point registered by the GPS and the “finish flag” represents the last registered data point by the GPS.

### Data processing

**Fig. 4:**
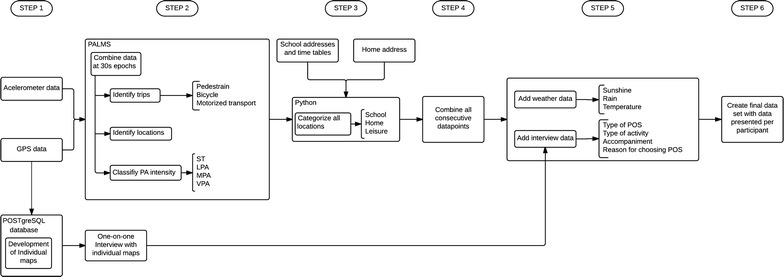
Data processing. *GPS* global positioning system, physical activity = physical activity, sedentary time = sedentary time, *LPA* light-intensity physical activity, *MPA* moderate-intensity physical activity, *VPA* vigorous-intensity physical activity, *POS* public open space

An overview of the data processing can be found in Fig. 4. First, all GPS and accelerometer data were created as CSV (comma separated value) files and imported into the Personal Activity and Location Measurement System (PALMS©) which was developed by the Centre for Wireless and Population Health Systems, University of California, San Diego.

Secondly, PALMS was used to merge all corresponding GPS and accelerometer data points (i.e. all data points-in epochs of 30 s-were matched according to the timestamp). PALMS identified speeds above 130 km/h, changes in distance higher than 1 km and elevations higher than 100 m between two data points (that are 30 s apart) as invalid data. In PALMS every data point (i.e. corresponding with an epoch of 30 s) was categorized into either an event or a transport related data point according to the acceleration measured. The transport related data points were further categorized into pedestrian (≥ 1 km/h < 10 km/h), bicycle (≥ 10 km/h, < 25 km/h) or motorized transport (≥ 25 km/h) [72] (data not reported). All data points that were not identified as transport, were categorized as an event. Additionally, all epochs were classified according to the physical activity intensity using Evenson cutpoints [67].

Thirdly, the PALMS dataset was combined with information on the home and school addresses and school time tables in Python. All data points that were identified as an event (i.e. not a trip) were categorized into three domains: school, home or leisure. The data were categorized in the domain school during school hours, when the participant was located at school (100 m buffer). Within the domain school, a distinction was made between physical education classes, other classes and recess based on the time tables of the participating classes. The home domain was defined as being at the home address with a 100 m buffer around the home. All other data were categorized in the leisure domain. A similar approach was used in previous Danish research [68].

Fourth, all consecutive data points allocated to the same domain were combined, resulting in a database with data per trip and event.

In the fifth step, all data from the individual interviews (i.e. for each POS location, the accompaniment, reason why they chose that POS and activities performed) and weather data (mean min sun/day, mean mm rain/day and average temperature/day) were added to the database. All trips or locations misclassified by PALMS were corrected using the interview data (e.g., when a participant indicated that a certain trip was done by bus, however, due to traffic congestion the speed was rather low (< 25 km/h) and this trip was falsely allocated to the bicycle category by PALMS, this was picked up during the interviews and corrected).

In order to perform the analyses, the data had to be presented per participant (instead of per event, as was the case after step five). Therefore, in the final data processing step, data were extracted from the data file created in step five, in order to create a new data file with data per participant. New variables were created with following information: mean wear time; mean number of POS visits accompanied by friends/classmates, siblings/cousins, parents/grandparents, organisation or alone; average sedentary time/day, in LPA/day, MVPA/day and VPA/day in total, located in POS (inclusive LPA, MVPA and VPA accumulated during trips to and from POS).

In this study, only time spent in the “leisure” category in POS and transportation to and from a POS was included. In other words, when a participant went to a park by bike, the time on the bike and the time spent in the park was included in the analyses. However, when a participant went to school by bike and cycled through a park, this trip was not included as this was categorized as a trip to school (and not POS) using active transportation.

### Data analysis

Descriptive statistics were calculated using IBM SPSS statistics 22 software. Chi^2^ tests and independent sample *t* tests were performed in SPSS to calculate differences between included and excluded participants (based on valid data).

Associations of individual factors (i.e. age, gender, ethnicity, education and sport club membership) and social environmental factors (accompaniment in POS with friends/classmates, siblings/cousins, parents/grandparents, organisation or alone) with the outcome measures (time, sedentary time, LPA, MVPA and VPA in POS, inclusive trips to and from POS) were examined using Multilevel Hurdle models and Gamma models (level 1 = subject, level 2 = school) using the package lme4 [73] in R version 3.4.1.

Different statistical models were used for the different outcomes as data were distributed differently. The outcome ‘time spent in POS’, was positively skewed and contained a high number of zeros (i.e. when a participant did not use a POS) demanding a multilevel hurdle model. A hurdle model includes two parts, first associations between the independent variables and the odds of having visited a POS were estimated by means of logistic regression analysis (binomial variance and logit link function) among all participants (n = 173). Second, a multilevel regression model with gamma variance and log link function was used to estimate the associations between the independent variables and the amount of time that was spent in POS among the participants who had used a POS (n = 130). The exponentiated regression coefficients represent the proportional difference in min spent in POS with a one-unit difference in the independent variables.

For the outcomes sedentary time, LPA, MVPA and VPA in POS, only the participants who had used a POS during data collection (n = 130) were included. This was done because participants who did not use a POS, logically also did not engage in any sedentary time, LPA, MVPA or VPA in POS. The outcomes ‘sedentary time in POS’, ‘LPA in POS’ and ‘MVPA in POS’, were skewed but did not contain many zeros and, therefore, multilevel regression models with gamma variance and log link function (selected based on Akaike’s Information Criterion) were fitted. These models estimate the association between the independent variables and the amount of time spent in sedentary time, LPA and MVPA in POS among the participants who had used a POS for sedentary time, LPA and MVPA. For the outcome VPA, a multilevel hurdle model was selected as data were skewed and contained a high number of zeros.

A stepwise procedure was used to build the models. First, all potential covariates (residence-urban, suburban or rural-, mean wear time, mean POS visits/day, number of days with valid data, rain, sun, temperature, total time in POS, mean min sedentary time/day for outcome sedentary time in POS, mean min LPA/day for outcome LPA in POS, mean min MVPA/day for outcome MVPA in POS and mean min VPA/day for outcome VPA in POS) were entered simultaneously into a model to identify those that were significantly related to the outcomes. Based on this, residence, temperature, mean wear time, mean POS visits/day, and number of days with data were included as covariates in all subsequent analyses. Mean min LPA/day, MVPA/day and VPA/day were included as covariates in the analyses with the outcome variables LPA, MVPA and VPA in POS, respectively, and total time in POS was included as a covariate in the analyses with the outcome variables sedentary time, LPA, MVPA and VPA in POS. Second, all individual factors (i.e. age, gender, ethnicity, education and sport club membership) were entered separately into a model adjusted for the appropriate covariates (see above).

Third, all individual factors that were significantly related to the outcome in the previous step were entered together into one model, again adjusting for the relevant covariates.

In a fourth step, each social environmental variable was entered separately into a model adjusting for the significant individual factors identified in step 2 and the relevant covariates. These four steps were performed separately for each outcome variable.

POS visitation in the company of an organisation was not included in the analyses, as only 2.5% of all POS visits were done in the company of an organisation. It was not possible to analyse associations between the environmental factors (i.e. location: street, shopping street/mall, square, park, outdoor sports ground/playground, parking lot, vacant lot and public transportation station/stop) and the outcome variables (SB, LPA, MVPA, VPA), because more than 70% of all POS visits were located at a public transportation stop/station. Level of significance was set at α = 0.05.

## Results

### Descriptive statistics

**Fig. 5:**
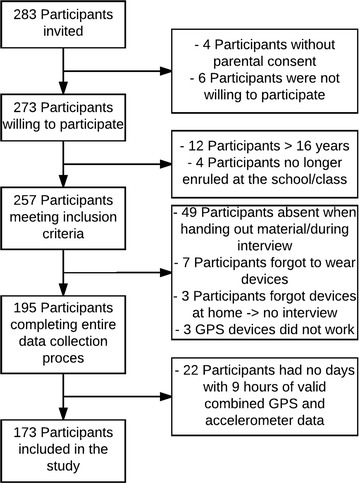
Sampling of the participants

In total, 283 adolescents were invited to participate in the study of which ten had no consent from their parents or were not willing to participate themselves. Of the remaining 273 participants, 100 were excluded from the analyses. Reasons for exclusion were: absence when handing out material or during interview (n = 49), no days with valid data for at least 9 h (n = 22), being older than 16 years (n = 12), forgot to wear material (n = 7), no longer enrolled at this school/class (n = 4), material forgotten at home (n = 3) or the GPS did not work properly (n = 3). Eventually, 173 participants aged 12–16 years were willing to participate, had parental consent and valid data for at least 1 day (Fig. 5).

No differences were found for gender, SES and ethnicity between the participants who were included for analysis (n = 173) and those who were excluded (n = 100) (*p* > 0.05). The excluded participants were significantly older than the included participants (15.6 vs. 14.2; *p* < 0.05) because participants older than 16 years were excluded from analyses.

The sample had a mean age of 14.2 ± 1.1 years, consisted of 54.4% girls and 93.1% was living in an urban or suburban environment. Most participants were enrolled in general education (68.8%), 28.3% had a non-western-European ethnicity and 22.5% had a lower SES based on parental educational level. Almost 60% of the participants were member of a sport club and the median min of MVPA/day was 36.5. Among the participants who used a POS (75.1% of the participants), the mean number of POS visits per day was 1.8 ± 1.2 (Table 1).

**Table 1:** Descriptive characteristics of the sample (n = 173)

Age (mean ± SD)	14.2 ± 1.1
Gender (% girls)	54.4
Living environment (%)	
Rural	6.9
Sub-urban	16.8
Urban	76.3
Education (%)	
General	68.8
Vocational	22.0
Technical	9.2
Other ethnicity (%)	28.3
Lower SES (%)	22.5
Sport club membership (%)	58.0
Sedentary time (mean h/day ± SD)	8.8 ± 1.6
LPA (mean h/day ± SD	3.3 ± 1.0
MVPA (median min/day; Q1, Q3)	36.5; 22.9, 51.4
% of participants who used a POS	75.1
Mean number of POS visits among participants who used a POS	1.83 ± 1.2

Skewed data were reported as median and interquartile range

*SD* standard deviation, *SES* socio-economic status, *MVPA* moderate- to vigorous-intensity physical activity, *Q1* 25th percentile, *Q3* 75th percentile, *min* minutes, *POS* public open space

All participants with one (n = 63) or 2 days (n = 110) of complete GPS and accelerometer data for 9 h minimum/day were included in the study. During the 283 included days 373 events took place at an outdoor POS. Participants reported that more than half of the POS visits were done in the company of a friend/classmate (59.8%) and most POS visits were located at a public transportation stop/station (71.0%). The most frequently mentioned reasons to visit a specific POS were: to wait for something/someone here (e.g., train) (30.3%), because friends/classmates/siblings/cousins wanted to go to that POS (17.4%), for ‘other reasons’ (e.g., for shopping purposes, easy to meet up) (17.4%) or because the POS was close to school or their home (13.8%). Standing was most frequently reported by the participants as the main activity in POS during a POS visit (43.1%), followed by walking (38.5%) and sitting or lying down (13.8%). The one-on-one interviews revealed that participants often indicated to ‘just hang around’ in POS while talking to friends (Table 2).

**Table 2:** Descriptive characteristics of POS visits (n = 373)

Company (% of POS visits; multiple answers possible)	% (n = 373)
Friends/classmates	59.8
Siblings/cousins	16.4
Parents/grandparents	16.4
Alone	15.6
Organisation	2.5
Location (% of POS visits)	
Public transportation stop/station	71.0
Street	9.4
Parking lot	5.4
Square	3.5
Shopping street	3.2
Sport field/playground	2.9
Park	2.9
Shopping mall	1.3
Vacant lot	0.3
Reasons for POS visit (% of POS visits; multiple answers possible)	
I had to wait for something/someone here (e.g., train)	30.3
My friends/classmates/siblings/cousins wanted go there	17.4
Other (e.g., for shopping purposes, easy to meet up)	17.4
This POS is close to my home/school	13.8
I was going somewhere else and decided to stay there	10.1
It is a habit to go there	8.3
There is a nice atmosphere	4.6
My parent want me to go there/I am not allowed to go anywhere else	4.6
There is sport infrastructure available	4.6
This POS is easy accessible	3.7
I know this place for a long time and I am familiar with this POS	1.8
Activity in POS (self-reported; multiple answers possible)	
Standing	43.1
Walking	38.5
Sitting/lying down	13.8
Ball sports	6.4
Biking	2.8
Other	1.8
Skateboarding/BMX/roller-skating	0.9
Active games	0.9
Jogging	0.9

*POS* public open space

### Associations of individual factors and company with time spent in pos

The logistic regression model shows that the odds for having used a POS were 2.20 times higher for participants with a non-western-European ethnicity compared to participants with a western-European ethnicity and 8.09 times higher for participants enrolled in technical education compared to participants enrolled in general education (both trends towards significance, see Table 3). In the multivariate model (data not shown in table), education became significant (OR: 8.68; 95% CI 1.03–72.75) while ethnicity remained borderline significant (OR: 2.33; 95% CI 0.93–5.86). For the participants who had visited a POS at least once during the days that were measured, results showed that with each additional visit accompanied by siblings, on average 60% more time was spent in POS per day (Exp. B: 1.60; 95% CI 1.26–2.04; data not shown in table). In other words, the higher the number of POS visits with siblings, the higher the total time spent in POS daily.

**Table 3:** Associations between individual factors and time spent in POS

Individual factors	Logistic regression^a^		Gamma model^b^	
	OR	95% CI	Exp. B	95% CI
Gender (ref = male)	1.82	0.88–3.79	0.98	0.69–1.38
Education (ref = general)				
Vocational	1.09	0.41–2.88	1.42	0.65–3.11
Technical	8.09°	0.97–67.62	1.15	0.61–2.15
Age	1.00	0.70–1.43	1.05	0.88–1.26
Ethnicity (ref = Belgium)	2.20°	1.88–5.49	1.25	0.84–1.86
Sport club membership (ref = yes)	1.80	0.85–3.85	1.21	0.87–1.68

O*R* odds ratio, *CI* confidence interval, *Exp. B* exponent of B, *POS* public open space, *ref* reference category, *min* minutes, ° α < 0.1 = trend towards significance

^a^The logistic regression model estimated the association of the independent factors with the odds of having visited a POS

^b^The Gamma models (Exp. B) estimated the proportional difference in min spent in POS associated with a one-unit difference in the independent variables for adolescents that had visited a POS. Analyses were controlled for mean temperature, residence, POS visits/day, total wear time (mean min/day), and amount of days. All Gamma models were fitted using the log link function

### Associations of individual factors and company with sedentary time and physical activity in pos

None of the individual and social environmental factors was significantly associated with sedentary time and LPA in POS (see Table 4). The analyses for the outcome MVPA in POS revealed that among the participants who had used a POS, girls engaged on average in 43% less min of MVPA/day in POS compared to boys. None of the other individual or social environmental factors were significantly associated with MVPA in POS.

**Table 4:** Associations between individual and social environmental factors with sedentary time, LPA and MVPA spent in POS

Individual factors	Gamma model sedentary time		Gamma model LPA		Gamma model MVPA	
	Exp. B	95% CI	Exp. B	95% CI	Exp. B	95% CI
Gender (ref = male)	0.89	0.63–1.27	0.73	0.53–1.00	0.57**	0.41–0.80
Education (ref = general)						
Vocational	1.43	0.85–2.40	0.71	0.42–1.19	0.74	0.44–1.24
Technical	1.11	0.62–2.00	0.93	0.51–1.67	0.72	0.40–1.29
Age	1.08	0.91–1.29	0.98	0.82–1.16	0.96	0.80–1.15
Ethnicity (ref = Belgium)	1.12	0.78–1.61	1.09	0.74–1.60	0.96	0.64–1.42
Sport club membership (ref = yes)	0.94	0.66–1.36	0.75	0.52–1.09	0.83	0.57–1.19

The Gamma models (Exp. B) estimated the proportional difference in sedentary time, LPA and MVPA in POS associated with a one-unit difference in the independent variables for adolescents that had used a POS. Analyses were controlled for mean temperature, residence, POS visits/day, total wear time (mean min/day), total time in POS and amount of days. All Gamma models were fitted using the log link function

*LPA* light-intensity physical activity, *MVPA* moderate- to vigorous-intensity physical activity, *OR* odds ratio, *CI* confidence interval, *Exp. B* exponent of B, *POS* public open space, *ref* reference category, *min* minutes, ° = α < 0.1 = trend towards significance

**α < 0.01

The logistic regression model for the outcome VPA in POS shows that girls had a 79% lower odds of having used a POS for VPA compared to boys and an increase in age with 1 year was associated with a 40% lower odds of having engaged in VPA in POS (trend towards significance for age, see Table 5). When gender (OR: 0.16; 95% CI 0.05–0.52) and age (OR: 0.52; 95% CI 0.30–0.93) were entered simultaneously into a model, both were significant (data not shown in table). Among those who had used a POS for VPA, girls engaged on average in 40% less min of VPA in POS/day compared to boys and participants enrolled in vocational education spent on average 41% less min in VPA in POS/day compared to participants enrolled in general education (trend towards significance for education). When gender and education were entered in the multivariable gamma model, only gender remained significant (Exp B: 0.63; 95% CI 0.41–0.98).

**Table 5:** Associations between individual and social environmental factors with VPA in POS

Individual factors	Logistic regression^a^		Gamma model^b^	
	OR	95% CI	Exp. B	95% CI
Gender (ref = male)	0.21**	0.07–0.63	0.60*	0.39–0.92
Education (ref = general)				
Vocational	0.70	0.17–2.90	0.59°	0.34–1.04
Technical	0.32	0.07–1.52	1.15	0.48–2.75
Age	0.60°	0.36–1.00	0.94	0.73–1.19
Ethnicity (ref = Belgium)	0.71	0.25–2.00	0.84	0.53–1.37
Sport club membership (ref = yes)	0.54	0.19–1.59	1.18	0.74–1.88

*VPA* vigorous-intensity physical activity, *OR* odds ratio, *CI* confidence interval, *Exp. B* exponent of B, *POS* public open space, *ref* reference category, *min* minutes, ° = α < 0.1 = trend towards significance

*α < 0.05; **α < 0.01

^a^The logistic regression model estimated the association of the independent factors with the odds of having used a POS for VPA

^b^The Gamma models (Exp. B) estimated the proportional difference in min of VPA in POS associated with a one-unit difference in the independent variables for adolescents that had used a POS. Analyses were controlled for mean temperature, residence, POS visits/day, total wear time (mean min/day), total time in POS, total time in VPA/day and amount of days. All Gamma models were fitted using the log link function

## Discussion

In this study, a socio-ecological approach was used to gain insight into the prevalence, frequency and context (i.e. company, locations and reason) of POS visitation and the factors associated with time, sedentary time and physical activity in POS among adolescents. Our study revealed that 75% of the participants used a POS and during most POS visits, participants were accompanied by friends/classmates. Mainly public transportation stops/stations were used, and subsequently the most reported reason for POS visitation was “to wait for something/someone (e.g., bus)”. Furthermore, ethnicity, education, gender and age were the individual factors associated with at least one outcome. The only social environmental variable associated with time spent in POS was accompaniment by siblings.

Surprisingly, there was limited variability in the POS locations used by the participants in this study as 70% of all POS visits were located at a public transportation stop/station. This suggests that public transportation stops/stations are frequently visited by adolescents in Flanders (Belgium), but these locations are not very suitable for physical activity. POS such as parks, a playground/sport field and squares are very suitable for physical activity, but were not often used by adolescents. Only 3.5% of the POS events was located at a square, 2.9% at a sport field/playground and 2.9% in a park. However, when the POS visits that took place at a public transportation stop/station are not taken into account, 12.0% of POS visits were located at squares; 10.3% at sport fields/playgrounds and 10.2% at parks. These findings are of importance for interventions aiming at the promotion of POS use among adolescents in Flanders, as we now know that POS such as parks, sport fields/playgrounds and squares are not often used and extra initiatives are warranted to encourage their use. Additionally, when public transportation routes are (re)designed, it is recommended to place public transportations stops close to locations suitable for physical activity (such as a park of square). Our results differ from previous Danish research where GPS measures revealed that 40% of the adolescents had used a playground, 97% had used urban green space and 32% had visited a shopping centre at 1 day during the data collection period [68]. It is difficult to compare the results of our study with these of this Danish study as the results are presented differently (i.e. % of events located at specific location, compared to % of participants that used a location), however, clearly some differences exist. On the one hand, some methodological differences between the studies could have caused these differences. In the Danish study, GIS was used to categorize the events into subdomains (i.e. locations such as playgrounds or urban green space) used during leisure time. It has been acknowledged that sometimes GIS layers lack details [45] which could have led to misclassification of events. For example, when a participant was waiting at the bus stop near a park, this could have been misclassified as an event in the park. Additionally, in the Danish study, the subdomain “public transportation stop/station” was not included, and 1–4 days of data were included whereas in our study only 1–2 days. On the other hand, these differences between studies could possibly be attributed to cultural differences between countries meaning that POS use is more integrated in Danish adolescents’ life [68].

This study provided new insight into the associations between the accompaniment and time, sedentary time and physical activity in POS. Results from the one-on-one interviews revealed that adolescents used POS most often with friends/classmates, followed by siblings, parents and alone. Previous research using ecological momentary assessment indicated that most 14-year-old adolescents reported to be physically active in the company of friends, followed by classmates and family members. Furthermore, the company with whom the greatest proportion of walking occurred was with friends or alone [74, 75]. In this study, only the accompaniment with siblings was associated with more time in POS, whereas no associations were found between the accompaniment and physical activity in POS. These contradicting results indicate that additional research on this topic is needed and that interventions targeting all children within a family could possibly be more effective. One explanation for this result could be that adolescents are allowed to stay longer outside when their parents know they are not alone, but in the company of a sibling.

It is known that total physical activity levels decline when adolescents grow older [76–78]. This study has added upon this knowledge by demonstrating that this age-dependent decrease also exist for POS physical activity. In this study, an increase in age with 1 year, was associated with a 40% lower odds of having engaged in VPA in POS. From previous qualitative research it became apparent that the playgrounds and facilities present in POS are often designed for younger children causing a lack of age appropriate facilities for (older) adolescents [27]. Creating POS with attractive facilities for older adolescents (such as sport fields [27] and adventurous playgrounds with high swings and big slides [79]) could possibly counteract this age-dependent decline in physical activity levels.

Total physical activity levels among adolescent girls have been shown to be lower than adolescent boys’ physical activity levels [77, 78, 80]. Additionally, our results revealed that also in POS, girls accumulate less physical activity compared to boys. Analyses revealed that boys spent more time in MVPA and VPA in POS compared to girls. This is in line with previous research from the US using GPS and accelerometers in a sample of 11- to 14-year-olds. It was reported that more physical activity was accumulated at playgrounds by boys compared to girls and boys had higher odds of spending time in MVPA at parks compared to girls [30]. Furthermore, previous observational research reported lower use of parks by girls (children and adolescents) and lower energy expenditure levels among girls compared to boys [26, 34, 81, 82]. Additionally, previous studies have shown that safety related factors (such as the presence of sufficient lighting [83], traffic safety [84], number of violent crimes [85]) were related to physical activity in parks and in the neighbourhood among girls. It is thus possible that safety issues contribute to gender differences in POS use. However, safety related factors are very context-specific and can differ between countries. In Belgium, the overall victimisation rate (= percentage of people victimised once or more) was significantly higher than the average of the 18 EU countries in 2004 [86].

Additionally, these results suggest that urban planners should consider adding attractive characteristics and features, in order to attract more girls to POS. It has been shown that adolescent girls prefer individual, non-competitive activities such as dancing or running or group activities with the focus on fun, such as netball [87–89]. Including features suitable for such activities could be a useful strategy to attract more girls to POS. However, additional research is needed to define what POS characteristics could specifically attract or repel girls for physical activity in POS.

Our study revealed ethnicity to be associated with time spent in POS among adolescents. The odds for having used a POS was higher among non-western-European adolescents compared to participants with a western-European ethnicity. However, it could be possible that adolescents with non-western-European ethnicity used public transportation more often, which could have influenced our results (because of the high number of POS visits that were located at public transportations stops/stations). This is an important result, as adolescents with a non-western-European ethnicity are often hard to reach for interventions. However, our results were only borderline significant and research on this topic among adolescents is lacking and, therefore, these results should be interpreted with caution.

Furthermore, this study revealed that participants enrolled in technical education were more likely to spent time in POS and participants enrolled in vocational education spent less min in VPA in POS compared to participants enrolled in general education. In Flanders (Belgium) technical education is focussed on practice lessons and technical-theoretical courses, whereas vocational education is focussed on learning a profession [90]. Not much is known about the association between education and time in POS among adolescents, but our findings are consistent with previous Australian research on adults’ individual factors associated with park use. This Australian study revealed that park users had less educational qualifications compared to non-park users [91]. However, adolescents enrolled in vocational education accumulated less min of VPA in POS compared to participants enrolled in general education. Currently, it is not known which POS characteristics invite adolescents to engage in VPA in POS and it is possible that differences exist according to educational level. Another explanation could be that adolescents enrolled in vocational education visit other types of POS what are less inviting for VPA (such as a train station). These findings have important social relevance as people with low educational level and low SES are at risk for low levels of physical activity [92] and are target populations that are hard to reach by standard physical activity initiatives from sport clubs or school sport. Therefore, interventions taking place in POS could have the ability to reach the target groups most in need for physical activity promotion. However, additional research is needed to define how adolescents could be encouraged to engage in physical activity in POS.

To our knowledge, this was the first study to look into the associations with sedentary time in POS. However, no associations were found with the individual nor with the social environmental factors. This could indicate that other factors are more important for sedentary time in POS. In this study, no environmental factors were included in the analyses, however, it is possible that the environmental characteristics of a POS (e.g., the presence of benches), are associated with sedentary time in POS. These factors should be included in future research.

This study emphasized the need for further research into the factors associated with time, sedentary time and physical activity in POS among adolescents. Within this study a social ecological approach was pursued. However, due to lacking variability in the POS locations that were used it was not possible to study the associations for the different types of POS locations that were used with time, sedentary time and physical activity in POS. Future studies could prevent this issue by assessing a larger sample from different cities and gathering data on more than 2 days. For larger samples, using data collected by the participants’ smartphones using mobile object trajectory analysis, could be a cost-effective and time-efficient option. Furthermore, it is recommended to develop a method in which subjective measurements can be obtained in a less time consuming manner. For example, using ecological momentary assessment via a smartphone application in combination with GPS and accelerometers could be a useful method [93]. Such an application can prompt questions about the accompaniment or about the characteristics of the public open space, when the smartphone detects that a participant is present at a public open space of interest. This way the use of a smartphone application could lessen the burden on the researchers and allow the researcher to collect data on more than 2 days. However, developing such an application poses some technical difficulties and is very expensive. In this study, no specific spatial analyses were performed such as spatial clustering or spatial time services. We suggest including such analyses in future research as these were outside the scope of this paper.

### Strengths and limitations

One of the major strengths of this study was the use of objective measurement methods for both locations and physical activity measures. By using these methods it was possible to investigate the locations that were actually used by the adolescents. Furthermore, these objectively measured data were combined with subjective interview data, to provide conclusive data and avoid the weaknesses of using solely qualitative or quantitative measurement methods [94]. Another strength was the broad definition of POS that was used in this study, whereas in other research often narrow definitions of POS were used. For example Edwards defined POS as “spaces reserved for the provision of green space and natural environments, accessible to the general public free of charge” and thereby excluded all non-green POS [95]. In this study, sedentary time and physical activity accumulated during trips to and from POS were included in analyses which, to our knowledge, has never been done before and provides a more comprehensive view on POS’ contribution to sedentary time and physical activity compared to previous studies that only included sedentary time and physical activity accumulated after arriving at the POS. Furthermore, it was attempted to include factors associated with POS use from different layers of the socio-ecological model in order to provide a more comprehensive insight into the use of POS. However, only individual and social environmental factors could be included into the analyses, because of the low levels of POS use and the low variability in POS locations that were used. This could be due to the fact that only 1 or 2 days of data were included for analyses, which was the biggest limitation of this study. Furthermore, also events that were more “transport” related (e.g., when participants were waiting for a bus at a bus stop, with the sole intention to take the bus) were included in our study and this could be considered as a limitation. Due to the structure of the data it was not possible to solely select the events located at a public transportation stop/station that could actually be classified as leisure time (e.g., when participants used a station as a meeting place). It is possible that the high number of POS visits located at public transportations stops/stations has altered the results. Another limitation of the study was that the data were collected from September to December, a period that is characterized by lower temperatures in this part of the world. This could have elicited different results compared to a period with generally better weather conditions. However, by including weather information (sun, rain and temperature) as covariates in the statistical analyses, we tried to tackle this barrier. Only three questions were included in the personal interviews and no questions were asked concerning the reasons for not engaging in physical activity. This could also be considered as a limitation of this study. The data were only collected in one city in Flanders (Belgium), inclusion of other cities could have provided different results and would have increased the generalizability of the current findings.

## Conclusion

Our research showed that ethnicity, education, gender, age and accompaniment are associated with time and physical activity in POS but not with sedentary time in POS among adolescents. Identifying the population groups that are currently least using POS (for physical activity) is important in order to guide interventions. In this study it was found that boys, younger adolescents, non-western-European adolescents and lower educated adolescents used POS more often (for physical activity). Additionally, the accompaniment by siblings in POS was shown to be associated with more time spent in POS. Understanding the use of POS is necessary in order to develop POS that are attractive to all adolescents and provide opportunities to engage in physical activity alone or in company. Additional research is warranted to elaborate on the current knowledge about the use of POS among adolescents.

## Additional file

**Additional file 1: Table S1.** Information about participating classes.

## Abbreviations

BMI: body mass index
CSV: comma separated value
GIS: geographical information system
GPS: global positioning system
LPA: light-intensity physical activity
MPA: moderate-intensity physical activity
MVPA: moderate- to vigorous-intensity physical activity
PALMS: personal activity and location movement system
POS: public open spaces
SES: socio-economic status
US: United States
VPA: vigorous-intensity physical activity
WHO: World Health Organisation
