# Supplementary material for: Factors related with public open space use among adolescents: a study using GPS and accelerometers
Source: Int J Health Geogr. 2018 Jan 22;17:3. doi: 10.1186/s12942-018-0123-2 (PMC5778634; doi:10.1186/s12942-018-0123-2)
Supplement: Supplementary file 1 — Additional file 1: Table S1. Information about participating classes. [file 12942_2018_123_MOESM1_ESM.docx]

## Additional file 1

Table 1: Information about participating classes

| Class | N | Study type | School year | % girls | age (mean ± SD) | Ethnicity (% other ethnicity) | SES (% Lower SES) |
| --- | --- | --- | --- | --- | --- | --- | --- |
| 1 | 12 | General | 3 | 83.3 | 14.1 ± 0.9 | 50 | 36.4 |
| 2 | 4 | General | 4 | 50.0 | 15.3 ± 0.5 | 25.0 | 0.0 |
| 3 | 16 | General | 1 | 81.3 | 12.2 ± 0.9 | 56.3 | 36.4 |
| 4 | 13 | General | 2 | 61.5 | 13.4 ± 1.0 | 76.9 | 25.0 |
| 5 | 9 | Technical | 3 | 100.0 | 14.1 ± 0.9 | 11.1 | 88.9 |
| 6 | 3 | Vocational | 4 | 66.7 | 14.5 ± 0.7 | 33.3 | 0.0 |
| 7 | 8 | Vocational | 4 | 75.0 | 15.3 ± 0.5 | 37.5 | 75.0 |
| 8 | 3 | Vocational | 4 | 33.3 | 15.7 ± 0.6 | 0 | 50.0 |
| 9 | 4 | Vocational | 4 | 66.7 | 15.5 ± 0.6 | 50.0 | 0.0 |
| 10 | 4 | Vocational | 4 | 100.0 | 15.3 ± 0.5 | 25.0 | 66.7 |
| 11 | 8 | General | 3 | 50.0 | 13.8 ± 0.5 | 12.5 | 0.0 |
| 12 | 17 | General | 4 | 52.9 | 15.0 ± 0.4 | 0.0 | 7.1 |
| 13 | 17 | General | 3 | 29.4 | 13.9 ± 0.4 | 23.5 | 6.7 |
| 14 | 6 | Technical | 4 | 50.0 | 15.7 ± 0.5 | 0.0 | 0.0 |
| 15 | 9 | Vocational | 3 | 77.8 | 14.1 ± 0.7 | 11.1 | 50.0 |
| 16 | 10 | Vocational | 4 | 0.0 | 15.4 ± 0.5 | 0.0 | 80.0 |
| 17 | 9 | General | 3 | 33.3 | 13.9 ± 0.3 | 22.2 | 33.3 |
| 18 | 21 | General | 3 | 28.6 | 14.2 ± 0.7 | 19.0 | 23.5 |

N = Number of participants in class, SD = Standard deviation, SES = Socio-economic Status
